# Clinical Efficacy in Skin Hydration and Reducing Wrinkles of Nanoemulsions Containing *Macadamia integrifolia* Seed Oil

**DOI:** 10.3390/nano14080724

**Published:** 2024-04-20

**Authors:** Suvimol Somwongin, Wantida Chaiyana

**Affiliations:** 1 Department of Pharmaceutical Sciences, Faculty of Pharmacy, Chiang Mai University, Chiang Mai 50200, Thailand; suvimol_s@cmu.ac.th; 2 Center of Excellence in Pharmaceutical Nanotechnology, Faculty of Pharmacy, Chiang Mai University, Chiang Mai 50200, Thailand; 3 Multidisciplinary and Interdisciplinary School, Chiang Mai University, Chiang Mai 50200, Thailand

**Keywords:** *Macadamia integrifolia*, hyaluronan, skin hydration, skin wrinkles, nanoemulsion, conventional emulsion

## Abstract

This study aimed to assess natural oils for their antioxidant and anti-hyaluronidase properties and select the most effective candidate for development into nanoemulsions (NE) for clinical evaluations. The oils were assessed using 2,2′-azino-bis(3-ethylbenzothiazoline-6-sulfonic acid (ABTS^•+^) and ferric thiocyanate assays for antioxidant properties and an enzyme-substrate reaction assay for anti-hyaluronidase activity. The most potent oil was formulated into conventional emulsions (CE) and NE, which were characterized and evaluated for their stability, both in accelerated and long-term conditions. The irritation potential was assessed using both the hen’s eggs chorioallantoic membrane test and a clinical trial. Skin hydration enhancement and skin wrinkle reduction efficacy were clinically assessed. *Macadamia integrifolia* oil exhibited significant potency as an ABTS^•+^ radical scavenger, lipid peroxidation inhibitor, and hyaluronidase inhibitor (*p* < 0.05). Both the CE and NE, comprising 15% *w*/*w* oil, 5% *w*/*w* Tween^®^ 80 and Span^®^ 80, and 80% *w*/*w* DI water, were found to be optimal. NE with an internal droplet size of 112.4 ± 0.8 nm, polydispersity index of 0.17 ± 0.01, and zeta potential of −31.5 ± 1.0 mV, had good stability and induced no irritation. Both CE and NE enhanced skin hydration and reduced skin wrinkles in human volunteers, while NE was outstanding in skin hydration enhancement.

## 1. Introduction

Emulsions play a crucial role in various industries, including food, pharmaceuticals, and cosmetics, as they enable the combination of typically immiscible substances like oil and water, with droplets dispersed within a continuous phase and separated by an interfacial boundary [1,2]. The structural and chemical consistency in traditional emulsions aids their production; however, this simplicity can constrain their range of applications [3]. Conventional or coarse emulsions, characterized by internal droplet sizes typically ranging from 0.1 to 100 μm, exhibit inherent thermodynamic instability because of their unfavorable interaction between oil and water molecules, ultimately resulting in their unavoidable breakdown over time [4]. Besides this, coalescence could happen when nearby droplets combine due to an increasing amount of compressive stress over time, which leads the interfacial film around the droplets starting to break down, initiating the coalescence process [5].

In contrast to conventional emulsions, nanoemulsions, characterized by droplets ranging from 20 to 200 nm, achieve stability through the interfacial layer of surfactants undergoing Brownian motion [2]. Nanotechnology, especially nanoemulsions, has gained growing interest from researchers across various industries. The cosmetic industry is recognized as a rapidly expanding sector, characterized by ongoing evolution through the adoption of new technologies and the integration of innovative and sustainable products [1]. Consequently, nanotechnology holds particular significance and has become an area of considerable interest and exploration. Nanoemulsions exhibit superior properties as a cosmetic base compared to conventional emulsions due to their ability to form smaller internal droplet sizes. These smaller droplets facilitate uniform distribution on the skin, enhance the delivery properties of active components, provide high stability, offer a larger surface area, and contribute to a pleasant aesthetic character and skin feel [6]. Moreover, these systems possess the potential to act as carriers for hydrophilic or lipophilic ingredients encapsulated within their internal droplets, subsequently allowing their release [7]. The prevalent nanoemulsion structure, known as oil-in-water (O/W), involves dispersed oil droplets within a continuous water phase, effectively encapsulating oil-soluble compounds. 

Therefore, this study aimed to develop and compare conventional O/W emulsions with O/W nanoemulsions using natural oils with cosmetic attributes. Furthermore, the clinical efficacy of the nano delivery systems in terms of skin hydration enhancement and skin wrinkle reduction was assessed, along with an evaluation of their safety.

## 2. Materials and Methods

### 2.1. Chemical Materials

*Macadamia integrifolia* (macadamia) seed oil, *Olea europaea* (olive) oil, *Triticum vulgare* (wheat) germ oil, *Persea americana* Mill. (avocado) oil, *Oenothera biennis* (evening primrose) oil, and *Prunus dulcis* Mill. (almond) oil were of cosmetic grade, and were purchased from Namsiang (Chiang Mai, Thailand). Polysorbate 20 (Tween^®^ 20), polysorbate 80 (Tween^®^ 80), polysorbate 85 (Tween^®^ 85), sorbitan oleate 80 (Span^®^ 80), butylene glycol, and hyaluronidase from bovine testes (EC 3.2.1.35) were of analytical grade, and were purchased from Sigma-Aldrich (St. Louis, MO, USA). Deionized water was purified using a Milli-Q water system (EMD Millipore, Burlington, MA, USA). Bovine serum albumin (BSA) was purchased from Merck (Darmstadt, Germany). Sodium acetate (CH_3_COONa), acetic acid (CH_3_COOH), hydrochloric acid (HCl), potassium persulfate (K_2_S_2_O_8_), sodium chloride (NaCl), monosodium phosphate (NaH_2_PO_4_), and disodium phosphate (Na_2_HPO_4_) were of analytical grade, purchased from RCI Labscan Co., Ltd. (Bangkok, Thailand).

### 2.2. Antioxidant Activities Determination of Natural Oils

#### 2.2.1. 2,2′-Azinobis (3-Ethylbenzothiazoline-6-Sulfonic Acid (ABTS) Assay

The ABTS^•+^ radical scavenging activities of each natural oil, including *M. integrifolia* oil, *O. europaea* oil, *T. vulgare* germ oil, *P. americana* oil, *O. biennis* oil, and *P. dulcis* oil, were assessed using methods slightly modified from Tachakittirungrod et al. (2007) and Chaiyana et al. (2019) [8,9]. In brief, each natural of the oils were added to an ABTS^•+^ solution, which was previously prepared and incubated overnight at a volume ratio of 1:9. Following incubation at room temperature for 5 min, the optical density was observed by a SPECTROstar Nano multimode detector (BMG Labtech GmbH, Ortenberg, Germany) set at 750 nm. The results have been presented in terms of Trolox equivalent antioxidant activity (TEAC). L-ascorbic acid served as the positive control. Triplicates of each experiment were run.

#### 2.2.2. Lipid Peroxidation Inhibition by the Ferric Thiocyanate (FTC) Assay

The lipid peroxidation inhibition of each of the natural oils, including *M. integrifolia* oil, *O. europaea* oil, *T. vulgare* germ oil, *P. americana* oil, *O. biennis* oil, and *P. dulcis* oil, was assessed using methods slightly modified from Chaiyana et al. (2017) [10]. In brief, each natural oil was added to the mixture containing 50% linoleic acid in DMSO, 5 mM NH_4_SCN solution, and 2 mM FeCl_2_ solution, at a volume ratio of 1:1. Following incubation at 37 ± 2 °C for 1 h, the optical density was observed using a SPECTROstar Nano multimode detector (BMG Labtech GmbH, Ortenberg, Germany) set at 500 nm. The results were presented as percentages of inhibition, which were calculated using the following equation:

Lipid peroxidation inhibition (%) = [(*A* − *B*)/*A*] × 100, (1)

where *A* is the optical density of the mixture without a sample and *B* is the optical density of the mixture with a sample. Trolox served as the positive control. Triplicates of each experiment were run.

### 2.3. Hyaluronidase Inhibitory Activity Determination of Natural Oils

The hyaluronidase inhibitory activities of each natural oil, including *M. integrifolia* oil, *O. europaea* oil, *T. vulgare* germ oil, *P. americana* oil, *O. biennis* oil, and *P. dulcis* oil, were assessed using methods slightly modified from Chaiyana et al. (2019) [9]. In brief, each natural oil was added to a mixture containing an enzyme diluent composed of 15 units/mL of hyaluronidase solution, 0.01% *w*/*v* BSA, and 77 mM NaCl in 20 mM phosphate buffer pH 5.35, at a volume ratio of 1:5. Following the incubation at 37 ± 2 °C for 10 min, 0.03% *w*/*v* hyaluronic acid in phosphate buffer pH 5.35 was added. After another incubation at 37 ± 2 °C for 45 min, the optical density was observed using a SPECTROstar Nano multimode detector (BMG Labtech GmbH, Ortenberg, Germany) set at 600 nm. The results have been presented as percentages of inhibition, which were calculated using the following equation:

Hyaluronidase inhibition (%) = [(*A* − *B*)/*A*] × 100, (2)

where *A* is the optical density of the mixture without sample and *B* is the optical density of the mixture with sample. Oleanolic acid served as the positive control. Triplicates of each experiment were run.

### 2.4. Determination of Required Hydrophilic Lipophilic Balance (RHLB) of Natural Oil 

A natural oil with the most remarkable cosmetic potential in terms of antioxidant and anti-hyaluronidase activities was selected for further investigations. To determine the RHLB of a natural oil, a series of surfactant mixtures with various HLB values were created for emulsion generation. Span^®^ 80 (HLB = 4.3) and Tween^®^ 80 (HLB = 15) were combined in different ratios to produce mixtures with HLB values ranging from 5 to 14. Subsequently, a 5% *w*/*w* surfactant mixture was utilized to create the emulsion through the conventional beaker method, with the oil and DI water each constituting 47.5% *w*/*w* of the emulsion. Upon the emulsion cooling to room temperature, the degree of phase separation was evaluated after each sample underwent centrifugation at 2000 rpm for 10 min. Aside from centrifugation, which was accelerated, the degree of phase separation was also determined after the emulsions were formed for 1, 7, and 30 days. 

### 2.5. Conventional O/W Emulsion Development

A selected natural oil was used to develop an O/W emulsion by blending Span^®^ 80 and Tween^®^ 80 in ratios corresponding to the RHLB of the oil. The selected natural oil at a concentration of 15% *w*/*w* was combined with a surfactant mixture ranging from 1 to 5% *w*/*w* to develop the conventional emulsions. O/W emulsions were prepared using the conventional beaker method. The oil phase, composed of the selected natural oil and Span^®^ 80, was heated up to 70 °C, while the aqueous phase, composed of DI water and Tween^®^ 80, was heated up to 75 °C. The oil phase was gradually added to the aqueous phase with continuous stirring. After the emulsion was formed, the resulting formulation was removed from the heat and left to cool to room temperature with continuous stirring. The conventional emulsion was kept in a closed container until further investigations. 

### 2.6. Characterizations and Stability Tests of Conventional O/W Emulsion

Conventional O/W emulsions underwent organoleptic inspections to evaluate their external appearance. Their pH was measured using a pH meter after being diluted 1:10 in DI water. The viscosity of each emulsion was assessed using a Brookfield rheometer equipped with a cone and plate geometry (R/S Rheometer, Brookfield Viscometer Ltd., Middleboro, MA, USA) at 25 °C. The stability of each emulsion was assessed following 6 cycles of heating–cooling. During each cycle, the emulsions were subjected to storage at 45 °C for 24 h, followed by storage at 4 °C for 24 h. In addition, a long-term stability test was also performed by storing the formulation at room temperature for 3 months. Subsequently, the physical appearance, pH, and viscosity were evaluated.

### 2.7. O/W Nanoemulsion Development

A selected natural oil was used to develop an O/W nanoemulsion using the combination of Span^®^ 80 and Tween^®^ 80 in proportions that matched the RHLB of the oil. Varying concentrations of the natural oil (ranging from 5 to 15% *w*/*w*) and a surfactant mixture (ranging from 5 to 15% *w*/*w*) were used to develop the nanoemulsions. The oil and Span^®^ 80 were combined to form an oil phase, while DI water and Tween^®^ 80 constituted the water phase. Subsequently, both oil and water phases were combined and underwent high-speed homogenization at 10,000 rpm for 5 min using a high-speed homogenizer (IKA^®^ T25 digital Ultra-Turrax, Staufen, Germany) to generate a primary emulsion, which then underwent 5 cycles of high-pressure homogenization at 500 bars using an APV 1000 homogenizer (APV 1000, Wilmington, MA, USA). The O/W nanoemulsion was kept in a sealed container until further investigations. 

### 2.8. Characterizations and Stability Tests of Nanoemulsions

O/W nanoemulsions underwent organoleptic inspections to evaluate their external appearance. The size, polydispersity index (PDI), and zeta potential were determined using a Zeta sizer instrument (Malvern Instruments Ltd., Malvern, UK). Results have been reported as the mean and standard deviation (S.D.) of at least ten measurements of each sample. In addition, their pH was measured using a pH meter. The viscosity of nanoemulsions was determined using a Brookfield rheometer equipped with a bob and cup (R/S Rheometer, Brookfield Viscometer Ltd., Middleboro, MA, USA) at 25 °C. The nanoemulsion morphology was analyzed using transmission electron microscopy (TEM), following the procedure outlined by Anantaworasakul et al. (2020) [11]. A single drop of nanoemulsion was placed onto a copper grid and stained with 1% phosphotungstic acid. TEM imaging was conducted at 100 kV to examine their nanostructure. The stability of each nanoemulsions was assessed following 6 cycles of heating–cooling. During each cycle, the nanoemulsions were subjected to storage at 45 °C for 24 h, followed by storage at 4 °C for 24 h. In addition, a long-term stability test was also performed by storing the formulation at room temperature for 3 months. Subsequently, the physical appearance, particle size, PDI, and zeta potential were evaluated.

### 2.9. Irritation Test

#### 2.9.1. In Vitro Hen’s Egg–Chorioallantoic Membrane (HET-CAM) Test 

The irritating potential of each nanoemulsion was assessed using the HET-CAM assay, following the methods outlined by Steiling et al. (1999) and Somwongin et al. (2018) [12,13]. The fertilized hen’s eggs, aged between 7 and 9 days, were kept in an automatic rotating mechanism set at 37.5 ± 0.5 °C and 62.5 ± 7.5% relative humidity. Before the investigation, the shell of each egg was carefully opened above the air cell using a rotating cutting blade attached to a Marathon-3 Champion dental micromotor (Saeyang, Daegu, Republic of Korea). The inner membrane in direct contact with the CAM was then moistened with 0.9% *w*/*v* sodium chloride solution (normal saline solution: NSS) and gently removed using forceps, ensuring no injury to the blood vessels. Subsequently, 30 µL of the nanoemulsions was applied to the CAM using a micropipette, and the time was noted as the start of exposure. Both positive control (1% *w*/*v* sodium lauryl sulfate solution) and negative control (NSS) were applied to the CAM to validate the experiment. Signs of irritation on the CAM, including vascular hemorrhage, lysis, and coagulation, were observed for up to 5 min under a stereo microscope (Olympus, Tokyo, Japan). The time of the first appearance of each irritation sign was recorded in seconds and used to calculate the irritation score (*IS*) using the formula:

*IS* = [(301 − *H*) × 5]/300 + [(301 − *L*) × 7]/300 + [(301 − *C*) × 9]/300, (3)

where *H* represents the time of first vascular hemorrhage, *L* represents the time of first vascular lysis, and *C* represents the time of first vascular coagulation. The *IS* values were classified as follows: 0.0–0.9, non-irritation; 1.0–4.9, slight irritation; 5.0–8.9, moderate irritation; and 9.0–21.0, severe irritation. Additionally, the blood vessels were re-examined for reactions after 1 h of exposure to the CAM. The experiments were conducted in duplicates.

#### 2.9.2. In Vivo Human Patch Test 

The in vivo human patch test conducted in this study received approval from the Human Research Ethics Committees at the Faculty of Pharmacy, Chiang Mai University, under the ethics approval number 001/2564. Thirty healthy volunteers participated in the examination, adhering to guidelines established by Basketter et al. (1997) [14]. All volunteers signed a consent form before participating to signify their agreement to be involved in the study. During the assessment, nanoemulsions were applied to the inner lower arm skin for 4 h. Skin irritation was monitored at 15, 30, 60, 120, 180, and 240 min intervals. After the completion of the 4 h of exposure, the skin was cleaned with water and dried with a wipe. The skin irritation was evaluated again after 24, 48, and 72 h.

### 2.10. Evaluations of Skin Hydration Enhancement and Skin Wrinkle Reduction in Human Volunteers

The identical group of thirty healthy volunteers who were engaged in the preceding in vivo human patch test were likewise involved in the efficacy study for skin hydration enhancement and skin wrinkle reduction. These volunteers continued to adhere to the guidelines established by Basketter et al. (1997) [14], ensuring consistency and maintaining ethical standards throughout the research process. Prior to assessing the formulation’s effectiveness in enhancing skin hydration and reducing wrinkles in human volunteers, participants were instructed to acclimate in a room for at least 15 min to allow their skin to adjust to the ambient conditions of 25 ± 1 °C temperature and 75 ± 2% relative humidity. On the first day, skin hydration was assessed on the inner lower arm of each volunteer using a Corneometer^®^ CM 825 (Courage and Khazaka Electronic GmbH, Cologne, Germany), while the topographies of the skin surface and skin wrinkles were measured from the photographs captured using a Skin Visiometer SV600 (Courage and Khazaka Electronic GmbH, Cologne, Germany). Various parameters were reported from the Skin Visiometer SV600, including skin volume, skin roughness (R1), maximum roughness (R2), average roughness (R3), smoothness depth (R4), and arithmetic average roughness (R5). Each volunteer received both the conventional O/W emulsion and O/W nanoemulsion for application on the inner lower arm, covering an area of 2 × 2 cm, twice daily (once in the morning and once before bedtime). After 30 days of applications, the skin was reassessed for skin hydration and skin wrinkles using a Corneometer^®^ CM 825 (Courage and Khazaka Electronic GmbH, Cologne, Germany) and a Skin Visiometer SV600 (Courage and Khazaka Electronic GmbH, Cologne, Germany), respectively. The efficacy of each formulation was calculated as follows:

Change in the skin parameter (%) = 100 × (B/A − 1) (4)

where *A* and *B* represents the parameters, including skin hydration, skin volume, R1, R2, R3, R4, and R5, before and after the application of each formulation for 30 days. The efficacies of both the conventional O/W emulsion and O/W nanoemulsion were compared with the control skin applied with nothing.

### 2.11. Statistical Analysis

The data have been reported as mean ± standard deviation (S.D.) from three independently performed experiments. Statistical analysis involving three or more groups was conducted using One-way ANOVA with SPSS Statistics 17.0 (IBM Corporations, New York, NY, USA), with significance set at *p* < 0.05. Conversely, statistical analysis for before and after treatments was determined by the paired *t*-test using SPSS Statistics 17.0 (IBM Corporations, New York, NY, USA), with significance levels denoted as * *p* < 0.05, ** *p* < 0.01, and *** *p* < 0.001.

## 3. Results and Discussion

### 3.1. Antioxidant and Hyaluronidase Inhibitory Activities of Natural Oils

**Figure 1:**
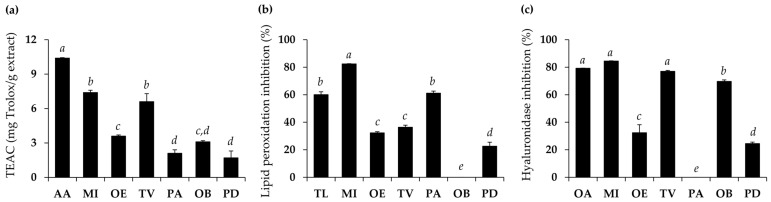
Antioxidant activities in terms of Trolox equivalent capacity (TEAC) (**a**) and lipid peroxidation inhibition (**b**), along with the hyaluronidase inhibitory activities (**c**) of ascorbic acid (AA), Trolox (TL), oleanolic acid (OA), and natural oils from *M. integrifolia* seed (MI), *O. europaea* (OE), *T. vulgare* germ (TV), *P. americana* (PA), *O. biennis* (OB), and *P. dulcis* (PD). Different letters (*a*, *b*, *c*, *d*, and *e*) denote statistical differences among the groups, determined through one-way ANOVA followed by the Tukey post hoc test (*p* < 0.05).

Natural oils were assessed for their antioxidant and hyaluronidase inhibitory activities, as presented in Figure 1. Antioxidant activities were evaluated in terms of Trolox equivalent antioxidant capacity (TEAC) to scavenge ABTS^•+^ radicals and lipid peroxidation inhibition. Among various natural oils, *M. integrifolia* oil and *T. vulgare* germ oil were found to be the most potent ABTS^•+^ radical scavengers, with the TEAC values of 7.4 ± 0.2 and 6.6 ± 0.7 mg Trolox per g extract, respectively (*p* < 0.05). On the other hand, *M. integrifolia* oil was found to exhibit the most potent lipid peroxidation inhibitory activity, with inhibition of 82.4 ± 0.2%, which is significantly higher than that of Trolox (60.1 ± 2.0%). Interestingly, *M. integrifolia* oil and *T. vulgare* germ oil were also found to display the most potent anti-hyaluronidase activities, with inhibitions of 84.5 ± 0.2% and 77.0 ± 0.7%, respectively (*p* < 0.05). Therefore, *M. integrifolia* oil was remarked as the most attractive natural oil with appealing radical scavenging, lipid peroxidation inhibitory, and anti-hyaluronidase properties. 

Oxidative stress is a primary contributor to skin aging, leading to the formation of wrinkles and other signs of aging [15]. Antioxidants play a vital role in skin rejuvenation by neutralizing harmful free radicals and reducing oxidative damage to skin cells [16,17]. Antioxidants have been reported to protect the skin against UV-induced damage [18], reduce collagen degradation [19], reduce inflammation [19], and thereby enhance the skin’s barrier function, resulting in more skin vitality and youthful-looking skin, as well as diminished visible signs of aging and maintenance. Hyaluronic acid has both structural and signaling roles [20]. Deficiencies in hyaluronic acid have been documented as a consequence of the aging process [21]. Both intrinsic and extrinsic factors contribute to the downregulation of hyaluronic acid synthases, leading to decreased levels of hyaluronic acid, particularly in the epidermis [22]. Human hyaluronidase, present both in organs and in body fluids, is responsible for the metabolism of hyaluronic acid [23,24]. Despite being widely distributed across various human tissues, hyaluronidase plays vital roles beyond hyaluronic acid degradation, particularly in skin aging, where its activity contributes to the loss of skin volume and elasticity, ultimately resulting in visible signs of aging such as wrinkles and sagging skin [25]. Therefore, the activity of human hyaluronidase is closely associated with the development of skin wrinkles and is a target for anti-aging interventions aimed at preserving hyaluronic acid levels in the skin. *M. integrifolia* oil, which possesses powerful antioxidant activities along with a significantly potent anti-hyaluronidase activity, is appealing for further use in the development of cosmetic and cosmeceutical formulations combatting skin wrinkles.

### 3.2. RHLB of M. integrifolia Oil

Regarding the potential of *M. integrifolia* oil in terms of antioxidant and anti-hyaluronidase activity, it was selected for further development in nanoemulsions. The RHLB value of *M. integrifolia* oil was investigated prior to its development since it is crucial in formulating emulsions and surfactant systems in terms of formulation compatibility, stability, uniformity, and desired emulsion characteristics [26,27]. Conventional emulsions containing *M. integrifolia* oil were formulated by combining different ratios of Span^®^ 80 (HLB = 4.3) and Tween^®^ 80 (HLB = 15) to achieve a range of HLB values between 5 and 14. The combination of Span^®^ 80 and Tween^®^ 80 was used in the current study due to their biodegradable properties, established safety profile, cost-effectiveness, and widespread availability [28,29,30]. Typically, Span^®^ 80 (sorbitan monooleate) is commonly paired with its polyethoxylated form, Tween^®^ 80, due to their analogous chemical structures, resulting in effective emulsion formation [29]. Besides this, the combination of Span^®^ 80 and Tween^®^ 80 has been reported for its notable efficacy in emulsion formation [29]. Furthermore, both emulsifiers are nonionic surfactants, which play a prominent role as surface-active agents in pharmaceuticals, cosmetics, and cosmeceuticals due to their significant advantages in terms of compatibility, stability, and toxicity compared to their cationic, anionic, or amphoteric counterparts [30]. According to their nonionic properties, they generally exhibit lower toxicity and reduced hemolytic activity, and cause less irritation to both skin and ocular surfaces, while also tending to maintain pH levels close to physiological values when dissolved [30].

**Figure 2:**
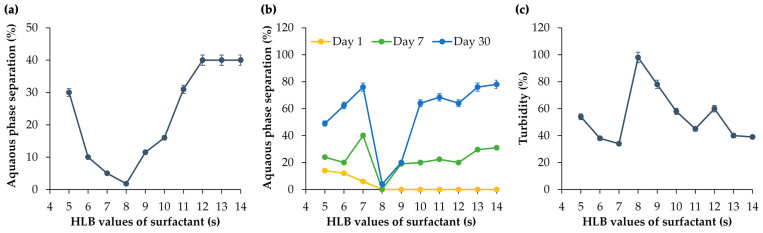
Aqueous phase separation after centrifugation (**a**) and formulation for 1, 7, and 30 days (**b**), as well as the turbidity (**c**) of emulsions developed using *M. integrifolia* oil and a series of Span^®^ 80 (HLB = 4.3) and Tween^®^ 80 (HLB = 15) mixtures with various HLB values ranging from 5 to 14.

It was clearly shown that at the HLB of 8, the least phase separation was detected after both centrifugation and long-term storage for 1, 3, and 7 days (Figure 2a,b). Similarly, consistent findings emerged in the turbidity measurement of the emulsion. The formulation using a surfactant mixture yielding an HLB of 8 was found to be the most stable, with the highest turbidity of 98% (Figure 2c). Therefore, it could be concluded that the RHLB of *M. integrifolia* oil was 8.

### 3.3. Conventional O/W Emulsions of M. integrifolia Oil

Conventional O/W emulsions of *M. integrifolia* oil were successfully developed when the concentration of the surfactant mixture was 5% *w*/*w*. The emulsion was white, opaque, creamy, and semisolid, with a viscosity of 1.75 ± 0.11 mPas and a pH of 6.28 ± 0.01, as shown in Table 1. In contrast, phase separations were detected in the formulations using 1 and 3% *w*/*w* of the surfactant mixture, indicating the instability of the emulsions. Therefore, they were excluded from the further stability study. The conventional O/W emulsions of *M. integrifolia* oil containing 5% *w*/*w* of the surfactants were found to be physically stable after six cycles of heating and cooling and 3 months of storage at room temperature, so they remained homogeneous with the same physical appearance. Nonetheless, pH values were significantly lower during both heating–cooling and long-term storage, while viscosity only increased after 3 months of storage (Table 1).

### 3.4. O/W Nanoemulsions of M. integrifolia Oil

**Figure 3:**
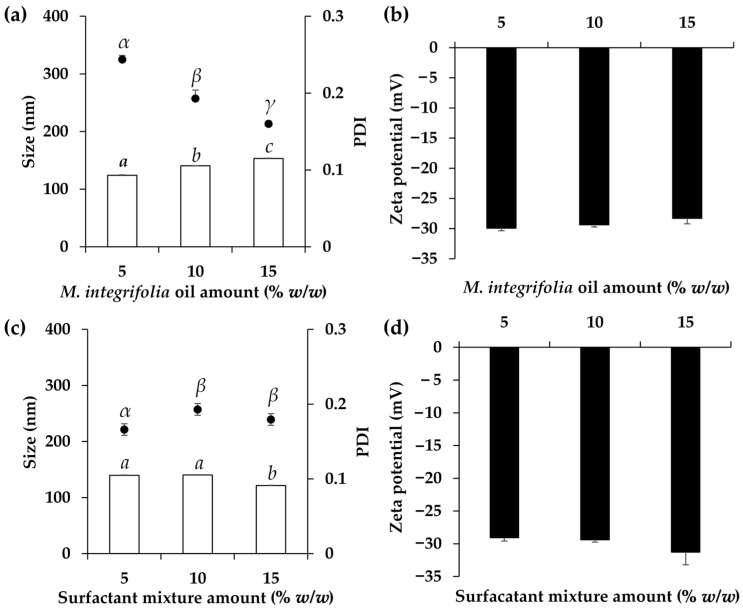
The internal droplet size and polydispersity index (PDI) (**a**) and zeta potential (**b**) of *M. integrifolia* oil nanoemulsions developed using various concentrations of oil and internal droplet size and PDI (**c**) and zeta potential (**d**) of *M. integrifolia* oil nanoemulsions developed using various concentrations of surfactant mixture. Different letters (*a*, *b*, and *c*) and Greek alphabet (α, β, and γ) denote statistical differences among the groups, determined through one-way ANOVA followed by the Tukey post hoc test (*p* < 0.05).

O/W nanoemulsions containing *M. integrifolia* oil were successfully formulated, but their characteristics varied depending on the compositions. Various oil concentrations had a significant impact on the internal droplet size and their polydispersity, while no observable effect was noted on the zeta potentials (Figure 3). Higher concentrations of *M. integrifolia* oil resulted in a larger internal droplet size, increasing from 124.1 ± 0.9 to 140.5 ± 0.6 and 153.4 ± 0.4 nm when the oil phases were 5, 10, and 15% *w*/*w*, respectively. The findings suggest that the developed nanoemulsions were indeed O/W nanoemulsions, as evidenced by the increase in internal droplet size with higher concentrations of the oil phase. Although the internal droplet size of the nanoemulsion with the *M. integrifolia* oil concentration of 15% *w*/*w* was found to be the largest, the size was still on the nanoscale, and it was able to be used as a delivery system. The previous study of Su et al. (2017) reported that nanoemulsions with a particle size of approximately 200 nm displayed transdermal permeation effects [31]. However, smaller nanoemulsions, e.g., 80 nm, were capable of diffusing into the viable epidermis without skin penetration, whereas larger nanoemulsions around 500 nm were unable to penetrate the stratum corneum and instead migrated along hair follicle canals [31]. Therefore, the nanoemulsions of *M. integrifolia* oil in a size range of 124.1 ± 0.9 to 153.4 ± 0.4 nm in the present study showed promising skin penetration through the stratum corneum. Aside from the alteration to the internal droplet size, the increase in the oil phase concentration led to a decreased PDI, from 0.24 ± 0.01 to 0.19 ± 0.01 and 0.16 ± 0.00 when the oil phases were 5, 10, and 15% *w*/*w*, respectively. As PDI is a measure of the uniformity of particle size distribution in a sample, with lower values indicating a more uniform distribution and higher values indicating greater variability in particle sizes, the nanoemulsions containing a higher concentration of *M. integrifolia* oil tend to have narrower PDI values, suggesting a more uniform distribution and less variability in particle sizes [32]. The concentration of *M. integrifolia* oil of 15% *w*/*w* was selected for further study due to its acceptable internal droplet size, narrowest PDI, and high content of *M. integrifolia* oil.

The effects of the concentration of the surfactant mixture were also assessed, as illustrated in Figure 3. No difference in the internal droplet size was observed when the surfactant concentrations were below 10% *w*/*w*, but after reaching a concentration of 15% *w*/*w*, the internal droplet size significantly decreased. The decrease in internal droplet size of the nanoemulsion would be advantageous in terms of skin penetration and efficacy [7]. However, it was observed that the PDIs increased after higher surfactant concentrations were used. Along with the risk of skin irritation posed by using a large amount of the surfactant, the recommended concentration of the surfactant was 5% *w*/*w*, which could yield a nanoemulsion with the desired internal droplet size of 139.7 ± 0.8, PDI of 0.17 ± 0.01, and zeta potential of −29.1 ± 0.5.

**Figure 4:**
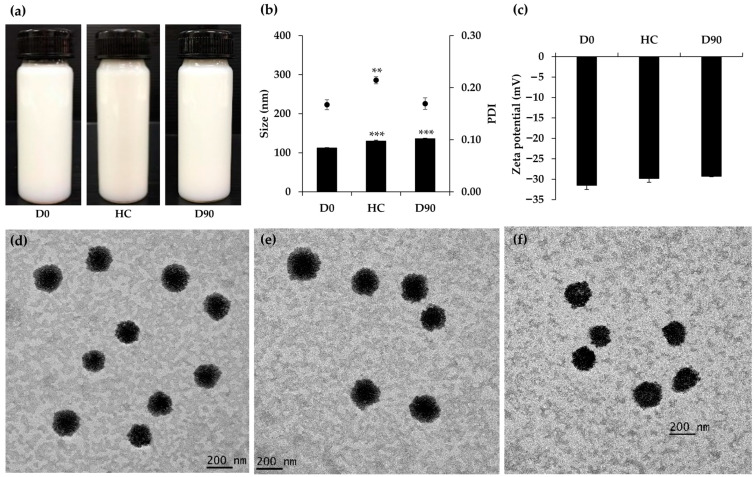
External appearance (**a**), internal droplet size and polydispersity index (PDI) (**b**), and zeta potential values (**c**) of *M. integrifolia* oil nanoemulsions before (D0) and after and accelerated stability test in 6 cycles of heating–cooling (HC) and long-term stability test at room temperature for 3 months (D90). Transmission electron microscopy (TEM) micrograph of *M. integrifolia* oil nanoemulsions before (**d**) and after and accelerated stability test in 6 cycles of heating–cooling (**e**) and long-term stability test at room temperature for 3 months (**f**). Asterisks denote significant differences among the groups (** *p* < 0.01 and *** *p* < 0.001) as determined by the paired *t*-test.

The stability of O/W nanoemulsions containing 15% *w*/*w* of *M. integrifolia* oil, 5% *w*/*w* of the surfactant mixture, and 80% *w*/*w* DI water was evaluated under six cycles of heating–cooling and during storage at room temperature for three months. The O/W nanoemulsion was observed to remain stable in terms of physical appearance and homogeneity (Figure 4a), with pH and viscosity also maintaining consistency after undergoing stability testing in both accelerated and long-term conditions (Table 2). Despite an increase in internal droplet size from 112.4 ± 0.8 nm to 130.1 ± 2.1 nm and 136.1 ± 1.2 nm after the accelerated and long-term stability tests, respectively, TEM images revealed that the particles retained their spherical shape, similar to their appearance before the stability tests (Figure 4d–f). Furthermore, particles were visually observed to be around 200 nm or smaller, as compared to the 200 nm bar size shown in Figure 4d–f. The findings of the TEM micrograph are consistent with the droplet size data obtained through the dynamic light scattering technique using a Zeta sizer instrument. The majority of the internal droplets in the nanoemulsion maintained a consistent shape and size throughout the 3-month long-term stability testing period, consistent with the PDI data showing an acceptable PDI of 0.17 ± 0.01. In contrast, TEM images revealed retained spherical internal droplets but with changes in size, corresponding to the higher PDI of 0.21 ± 0.01. However, the PDI, representing the size distribution within a sample, ranges from 0.0 (perfectly uniform) to 1.0 (highly polydisperse), with values ≤ 0.2 considered acceptable for polymer-based nanoparticles and ≤0.3 indicating homogeneity in lipid-based carriers for drug delivery [33]. Therefore, considering the acceptable internal droplet size and PDI, as well as the maintained physical appearance, TEM micrograph, and zeta potential values, the O/W nanoemulsion of *M. integrifolia* oil in this study was determined to be stable under both accelerated and long-term conditions.

### 3.5. Irritation Properties of Emulsion and Nanoemulsions of M. integrifolia Oil

**Figure 5:**
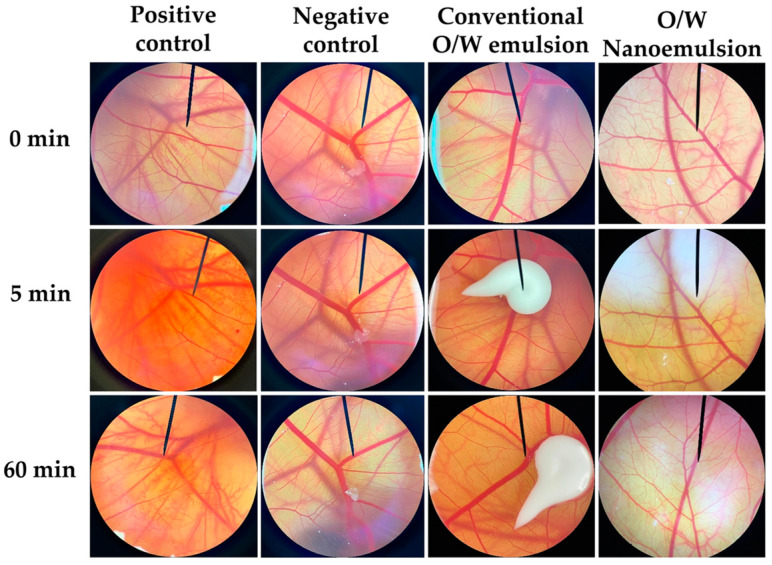
Photographs illustrating the effects of positive control (1% *w*/*v* sodium lauryl sulfate aqueous solution), negative control (0.9% *w*/*v* sodium chloride aqueous solution or normal saline solution), conventional O/W emulsion of *M. integrifolia* oil, and O/W nanoemulsion of *M. integrifolia* oil on the chorioallantoic membrane before exposure to the sample (0 min) and after exposure for 5 and 60 min.

Conventional O/W emulsion and O/W nanoemulsion formulations, comprising 15% *w*/*w M. integrifolia* oil, 5% *w*/*w* surfactant mixture, and 80% *w*/*w* DI water, were successfully developed. According to the potential of *M. integrifolia* oil in terms of its antioxidant activity and hyaluronidase inhibition, it holds promise for cosmetic or cosmeceutical applications. Consequently, conducting irritation tests is indispensable to ensuring product safety, regulatory adherence, consumer confidence, and liability mitigation during product development and market entry. Although testing on animals ensures product safety for humans, growing concerns for animal welfare have prompted legislation to minimize animal pain and injury [34,35]. Alternative methods, like the modified test using hen’s egg–chorioallantoic membrane, known as the HET-CAM assay, aim to reduce reliance on animal testing while ensuring product safety [36,37]. Besides this, the HET-CAM test utilized seven-day-old hen’s eggs, which were in an early stage of embryonic growth, falling within the initial phase of incubation and not necessitating ethical committee approval [38]. The CAM exposed to the aqueous solution of sodium lauryl sulfate was found to be irritated, exhibiting signs of vascular hemorrhage, lysis, and coagulation (Figure 5). The irritation was classified as severe, with an IS of 18.5 ± 0.1 (Table 3). In contrast, the negative control, represented by NSS, demonstrated no signs of irritation, consistent with the findings observed in both the conventional emulsion and nanoemulsion formulations containing *M. integrifolia* oil. The findings indicate the safety of both formulations, suggesting their suitability for further potential applications in the cosmetic or cosmeceutical areas.

Following the favorable results obtained from the irritation tests in HET-CAM, clinical trials in 30 human volunteers were conducted to evaluate the safety and tolerance of the *M. integrifolia* oil O/W emulsion and O/W nanoemulsion. The results reveal no signs of irritation or adverse reactions among the human participants, indicating the compatibility of both formulations with human skin. These findings provide further evidence supporting the safety profile of the formulations and suggest their potential suitability for cosmetic or cosmeceutical applications. Moreover, the absence of irritation in human subjects reinforces the promising results obtained from earlier tests, highlighting the overall safety and potential for use as both conventional emulsion and nanoemulsion in consumer products.

### 3.6. Skin Hydration Enhancement and Skin Wrinkle Reduction Properties

**Figure 6:**
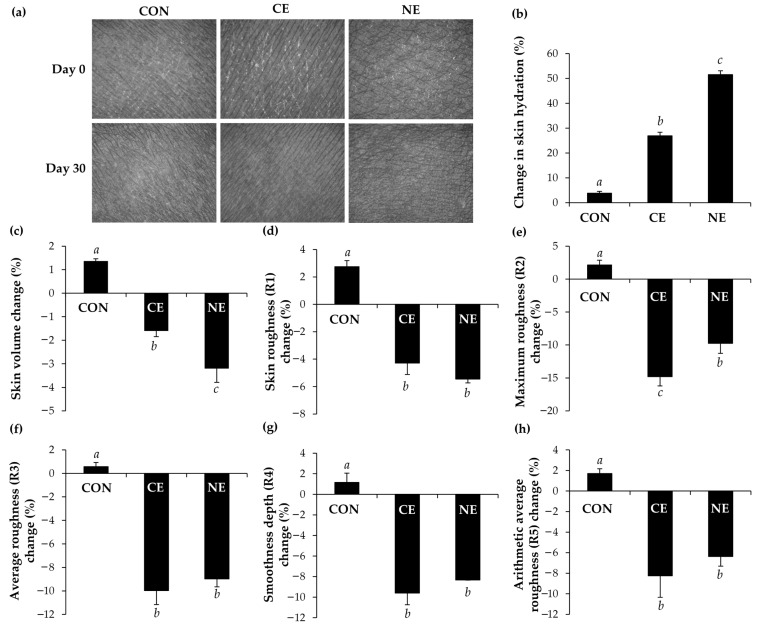
Photograph from Skin Visiometer SV600 illustrating the skin before (Day 0) and after being applied with nothing (CON) as well as conventional O/W emulsion of *M. integrifolia* oil (CE), and O/W nanoemulsion of *M. integrifolia* oil (NE), for 30 days (Day 30) (**a**), as well as the change in skin hydration (**b**), skin volume (**c**), skin roughness (R1) (**d**), maximum roughness (R2) (**e**), average roughness (R3) (**f**), smoothness depth (R4) (**g**), and arithmetic average roughness (R5) (**h**). Different letters (*a*, *b*, and *c*) denote statistical differences among the groups, determined through one-way ANOVA followed by the Tukey post hoc test (*p* < 0.05).

The effectiveness of both the *M. integrifolia* oil conventional O/W emulsion and O/W nanoemulsion was evaluated by comparing with a control group where no product was applied to the skin. The photographs from Skin Visiometer SV600 illustrating the topography of the skin before and after the application of *M. integrifolia* oil conventional emulsion and nanoemulsion, as shown in Figure 6a, highlight the increased moisture and smoothness of the skin. The findings are in line with the skin hydration evaluated using the Corneometer^®^ CM 825 (Courage and Khazaka Electronic GmbH, Cologne, Germany). The nanoemulsion containing *M. integrifolia* oil notably improved skin hydration by 51.5 ± 3.7%, a significant increase compared to the conventional O/W emulsion and the control, which demonstrated skin hydration enhancements of 26.9 ± 2.7% and 3.8 ± 2.3%, respectively. The significant enhancement in skin hydration is likely attributed to the occlusive effect of the small internal *M. integrifolia* oil droplets in the nanoemulsion, which can effectively cover the skin and reduce transepidermal water loss. The findings are consistent with the previous study by Pereira et al. (2022), which highlighted that the delivery of active ingredients via nanoemulsions offers a superior alternative to traditional cosmetics [39]. This is attributed to their enhanced hydration efficacy resulting from their nanosize, which improved their occlusive properties [39]. Jaslina et al. (2022) similarly reported that the peel-off O/W nanoemulsion containing kojic monooleate applied to the skin of human volunteers suggested an increase in skin hydration, likely due to its occlusive effect [40]. Barreto et al. (2017) also observed that the nanoemulsion comprising 40% oil phase, 50% aqueous phase, and 10% surfactants increased the water content of the stratum corneum by 10.13% compared to its vehicle and by 19.28% compared to its baseline values [40]. As the moisture content of the skin is one of the vital parameters affecting the skin structure, appearance, and its protective function [39], the nanoemulsion from *M. integrifolia* oil, which could improve skin hydration by 51.5 ± 3.7%, would be attractive for use in skincare formulations.

Aside from the skin hydration enhancement effect, *M. integrifolia* oil formulations were found to reduce the skin wrinkles and roughness (Figure 6). In brief, skin roughness (R1) signifies the difference between the highest and lowest peaks in wrinkles, with maximum roughness (R2) representing the largest R1 values and average roughness (R3) being the arithmetic average of R1 values within these sections [41]. The smoothness depth (R4) represents the average depth of skin wrinkles, calculated by dividing the area formed by the wrinkle roughness by the length of the horizontal line after the integration of the highest peak and horizontal line, while the arithmetic average roughness (R5) is determined by integrating the area formed by the middle line of wrinkle roughness and dividing it by the length of the middle line, indicating the arithmetic mean roughness [41]. These parameters are measured in arbitrary units (A.U.), with lower values indicating improved skin wrinkles [41]. The findings indicate that both the conventional emulsion and nanoemulsion of *M. integrifolia* oil could significantly reduce the skin volume and R1–R5, indicating uniform effects across multiple variables and reinforcing the reliability of the findings. However, the nanoemulsion of *M. integrifolia* oil outperformed the conventional emulsion in terms of skin volume reduction. The application of *M. integrifolia* oil nanoemulsion resulted in a significant reduction in skin volume by 3.2 ± 0.6%, whereas the conventional emulsion led to a reduction of 1.6 ± 0.2%. A previous study by Ryu et al. (2014) proposed a strong correlation between skin wrinkles, roughness parameters, and visual texture scores, indicating the potential utility of an established skin texture scoring index for evaluating skin aging [42]. Therefore, the nanoemulsion of *M. integrifolia* oil, which enhances skin hydration by 51.5 ± 3.7% and reduces skin volume by 3.2 ± 0.6%, R1 by 5.4 ± 0.3%, R2 by 8.0 ± 0.3%, R3 by 9.0 ± 0.7%, R4 by 8.3 ± 0.0%, and R5 by 6.4 ± 0.9%, would likely be beneficial for skincare and anti-aging applications.

## 4. Conclusions

*M. integrifolia* oil emerged as the most potent among various natural oils due to its remarkable radical scavenging properties (TEAC values of 7.4 ± 0.2 mg Trolox per g extract), lipid peroxidation inhibition (82.4 ± 0.2%), and hyaluronidase inhibition (84.5 ± 0.2%). Its lipid peroxidation inhibition was significantly higher than that of Trolox (60.1 ± 2.0%). Additionally, *M. integrifolia* oil was effectively formulated into both conventional emulsion and nanoemulsion compositions, comprising 15% *w*/*w* of *M. integrifolia* oil, 5% *w*/*w* of a mixture of Tween^®^ 80 and Span^®^ 80 with a resulting HLB of 8, and 80% *w*/*w* DI water. The resulting nanoemulsion displayed an internal droplet size of 112.4 ± 0.8 nm, a PDI of 0.17 ± 0.01, and a zeta potential of −31.5 ± 1.0 mV, with good stability during both accelerated and long-term storage periods of 3 months. All *M. integrifolia* oil formulations were found to be safe and induce no irritation in both the HET-CAM test and the clinical trial in human volunteers. Besides this, both formulations could enhance skin hydration and reduce skin wrinkles and roughness in human volunteers. The nanoemulsion containing *M. integrifolia* oil exhibited outstanding skin hydration enhancement. Therefore, *M. integrifolia* oil shows great potential for cosmetic and cosmeceutical applications, providing both antioxidant and anti-aging benefits.

## Figures and Tables

**Table 1:** Homogeneity, pH, and viscosity of conventional O/W emulsions of *M. integrifolia* oil before and after the stability tests.

Stability Test	Homogeneity	pH	Viscosity (mPas)
Before stability test	√	6.28 ± 0.01	1.75 ± 0.11
After 6 cycles of heating–cooling	√	6.13 ± 0.03 ***	1.85 ± 0.10
After 3 months in room temperature	√	6.21 ± 0.00 **	2.09 ± 0.09 *

NOTE: √ represents homogeneity. Asterisks denote significant differences among the groups (* *p* < 0.05, ** *p* < 0.01, and *** *p* < 0.001).

**Table 2:** Homogeneity, pH, and viscosity of O/W nanoemulsions of *M. integrifolia* oil before and after the stability tests.

Stability Test	Homogeneity	pH	Viscosity (mPas)
Before stability test	√	6.37 ± 0.02	0.21 ± 0.02
After 6 cycles of heating–cooling	√	6.21 ± 0.01 ***	0.16 ± 0.00 **
After 3 months in room temperature	√	6.32 ± 0.02 *	0.36 ± 0.00 ***

NOTE: √ represents homogeneity. Asterisks denote significant differences among the groups (* *p* < 0.05, ** *p* < 0.01, and *** *p* < 0.001) as determined by the paired *t*-test.

**Table 3:** Irritation score and severity of *M. integrifolia* oil O/W emulsion and O/W nanoemulsion.

Tested Compounds	Irritation Score (IS)	Severity
Positive control	18.5 ± 0.1	Severe irritation
Negative control	0.0 ± 0.0	No irritation
Conventional O/W emulsion	0.0 ± 0.0	No irritation
O/W Nanoemulsion	0.0 ± 0.0	No irritation

NOTE: Positive control = 1% *w*/*w* sodium lauryl sulfate aqueous solution and negative control = 0.9% *w*/*w* sodium chloride aqueous solution or normal saline solution.

## Data Availability

The data presented in this study are available from the corresponding author upon reasonable request.
